# Insight Into Seeded Tau Fibril Growth From Molecular Dynamics Simulation of the Alzheimer’s Disease Protofibril Core

**DOI:** 10.3389/fmolb.2021.624302

**Published:** 2021-03-19

**Authors:** Cass Leonard, Christian Phillips, James McCarty

**Affiliations:** Department of Chemistry, Western Washington University, Bellingham, WA, United States

**Keywords:** tau, Alzheimer's disease, neurofibrillary tangles, paired-helical filament, straight filament, umbrella sampling, molecular dynamics

## Abstract

Aggregates of the microtubule associated tau protein are a major constituent of neurofibrillary lesions that define Alzheimer’s disease (AD) pathology. Increasing experimental evidence suggests that the spread of tau neurofibrillary tangles results from a prion-like seeding mechanism in which small oligomeric tau fibrils template the conversion of native, intrinsically disordered, tau proteins into their pathological form. By using atomistic molecular dynamics (MD) simulations, we investigate the stability and dissociation thermodynamics of high-resolution cryo-electron microscopy (cryo-EM) structures of both the AD paired-helical filament (PHF) and straight filament (SF). Non-equilibrium steered MD (SMD) center-of-mass pulling simulations are used to probe the stability of the protofibril structure and identify intermolecular contacts that must be broken before a single tau peptide can dissociate from the protofibril end. Using a combination of exploratory metadynamics and umbrella sampling, we investigate the complete dissociation pathway and compute a free energy profile for the dissociation of a single tau peptide from the fibril end. Different features of the free energy surface between the PHF and SF protofibril result from a different mechanism of tau unfolding. Comparison of wild-type tau PHF and post-translationally modified pSer356 tau shows that phosphorylation at this site changes the dissociation free energy surface of the terminal peptide. These results demonstrate how different protofibril morphologies template the folding of endogenous tau in distinct ways, and how post-translational modification can perturb the folding mechanism.

## 1 Introduction

Alzheimer’s disease (AD) is characterized by extracellular plaque deposits of amyloid-β (Aβ) peptides and intracellular neurofibrillary tangles (NFTs) of the microtubule associated protein tau (Selkoe and Hardy, 2016; Polanco et al., 2018). Both Aβ and tau contribute to neuroinflammation and neurodegeneration (Bolós et al., 2017). Evidence suggests that Aβ and tau interact synergistically in AD pathogenesis (Rhein et al., 2009; Ittner and Götz, 2011), and amyloid plaques have been shown to facilitate the seeding of tau fibrils (He et al., 2018). As a complement to amyloid-β-based drugs for AD, tau is a potential target for therapeutics aimed at blocking tau aggregation (Noble et al., 2011; Li and Götz, 2017).

Tau is a soluble, intrinsically disordered protein (IDP) predominantly found in axons (Konzack et al., 2007). Under physiological conditions, tau binds to microtubules and plays an important role in microtubule stabilization, the regulation of active axonal transport, and neuronal polarity (Gonzalez-Billault et al., 2002; Götz et al., 2006). Full-length tau (2N4R) consists of 441 amino acids with a N-terminal region, a proline-rich domain, four (R1-R4) microtubule binding repeat (MBR) domains, and a C-terminal region. Alternative mRNA splicing produces six isoforms of tau in the human brain with either three (R1, R3, and R4) or four (R1-R4) microtubule binding repeat (MBR) domains. In AD NFTs, both the three- and four-repeat isoforms are present (Goedert and Spillantini, 2006). Tau has 84 available serine (S), threonine (T), and tyrosine (Y) phosphorylation sites, located primarily in the proline-rich and C-terminal domains (Goedert et al., 2017a). Hyperphosphorylation of tau at both physiological and pathological phosphorylation sites causes tau to dissociate from microtubules and is observed in both AD patients and transgenic mouse models (Ballatore et al., 2007).

It is hypothesized that the spread of tau pathology in the brain progresses via a prion-like mechanism, in which oligomeric tau or other aberrant pre-fibrillar species induces other tau molecules to adopt a particular pathological structure (Mudher et al., 2017; Goedert et al., 2017a; Goedert et al., 2017b; Ayers et al., 2018; Jucker and Walker, 2013). Both *in vitro* and *in vivo* experiments show that small, oligomeric tau complexes seed the growth of tau fibrils (Strang et al., 2018). This seed can be a small tau protofibril isolated from mouse or human brain tissue. Different structural seeds induce different tau fibril morphologies by recruiting tau in solution to polymerize onto the protofibril end, consistent with a prion-like hypothesis (Ayers et al., 2018). The precise mechanism by which endogenous tau is converted into a particular fibril structure is not well-understood, but likely depends on subtle differences in chemical environment, signaling, environmental stress, or mutations.

The predominant component of tau NFTs is a paired helical filament (PHF) structure formed by a twisted, double helical stack of C-shaped subunits (Kidd, 1963). A second structural polymorph, called the straight filament (SF), is also found in tau inclusions and consists of a similar C-shaped unit forming different lateral contacts between filament subunits (Crowther, 1991). Recent high-resolution cryo-EM structures of the tau fibril core, isolated from the brain of an individual with AD, reveal the detailed C-shaped core structure formed by residues 306–378 that are part of the R3-R4 repeat domain (Fitzpatrick et al., 2017). The C-shaped fibril core has a combined cross-β/β-helix structure typical of amyloid fibrils and prion structures (Sunde et al., 1997; Govaerts et al., 2004). The core residues include the PHF6 hexapeptide (_306_VQIVYK_311_ in R3) that has been identified as a minimal interaction motif for tau aggregation and amyloid formation (Friedhoff et al., 2000; Luo et al., 2014; Ganguly et al., 2015; Xie et al., 2015).

Molecular dynamics (MD) simulations can provide atomic-resolution information about the stability and thermodynamics of tau fibril elongation. For example, Li et al. performed all-atom, 100 ns MD simulations of the C-shaped motif, demonstrating that the form is stable only for the R3-R4 repeat domains while the R1-R2 adopts a linear shape (Li et al., 2018). MD simulations of full-length tau in solution reveal that tau samples both extended and compact conformations and can transiently form secondary structures resembling the fibril state (Battisti et al., 2012). All-atom replica exchange MD (REMD) simulations (Larini et al., 2013; Ganguly et al., 2015; Levine et al., 2015) and coarse-grained (Smit et al., 2017) simulations of important nucleating fragments of tau have provided information about the early stages of tau aggregation and which factors stabilize either parallel or antiparallel β-sheet structures (Ganguly et al., 2015). REMD simulations performed by Derreumaux et al. of the R3-R4 domain dimer identified elongated, U-shaped, V-shaped, and globular configurations, but not the C-shaped structure characteristic of AD NFTs (Derreumaux et al., 2020). Recent steered molecular dynamics (SMD) simulations assessed the stability and dissociation of tau from an isolated protofibril pentamer, suggesting that the PHF and SF protofibrils induce a different pathway for misfolding of tau (Liu et al., 2019).

Despite advances in processing power and designated custom hardware (Shaw et al., 2014), conventional atomistic MD simulations in explicit solvent of protofibril nucleation and elongation remain particularly challenging due to the large time scale and system sizes characteristic of protein aggregation. Enhanced sampling methods can overcome this challenge by accelerating the exploration of configurational state space through the use of an applied bias potential. Umbrella sampling (Torrie and Valleau, 1977) is particularly suited to compute the free energy surface (FES) along a pre-defined reaction coordinate (Roux, 1995). Umbrella sampling has been applied to study both the thermodynamics and kinetics of Aβ fibril growth, providing insight into the stability and formation of the Aβ fibril (Lemkul and Bevan, 2010; Schwierz et al., 2016).

In this work, we use all-atom molecular dynamics simulation in explicit solvent to study the paired PHF and SF protofibril structures and a post-translationally modified PHF, phosphorylated at residue Ser356. We assess the stability of the protofibril structure from SMD simulations, identifying the structural changes that occur in response to the applied force at its maximum value. The structural changes that result from the applied force reveal interchain interactions that impart stability to the fibril. We then perform an exploratory metadynamics simulation to determine the dissociation pathway of a single tau peptide “monomer” from the protofibril end. To obtain the free energy surface (FES) for tau dissociation, we perform umbrella sampling simulations of configurations sampled along the dissociation pathway, using a harmonic restraining potential to sample the configurational space along the COM distance reaction coordinate. The FES along the dissociation coordinate, obtained using the weighted histogram analysis method (WHAM), provides thermodynamic and mechanistic insight into the stability of tau protofibrils and the dissociation mechanism of a single tau peptide from the fibril end. This work can provide a foundation for designing and interpreting tau seeding experiments with different fibril morphologies and for designing small molecule inhibitors that destabilize tau NFTs.

## 2 Methods

**FIGURE 1:**
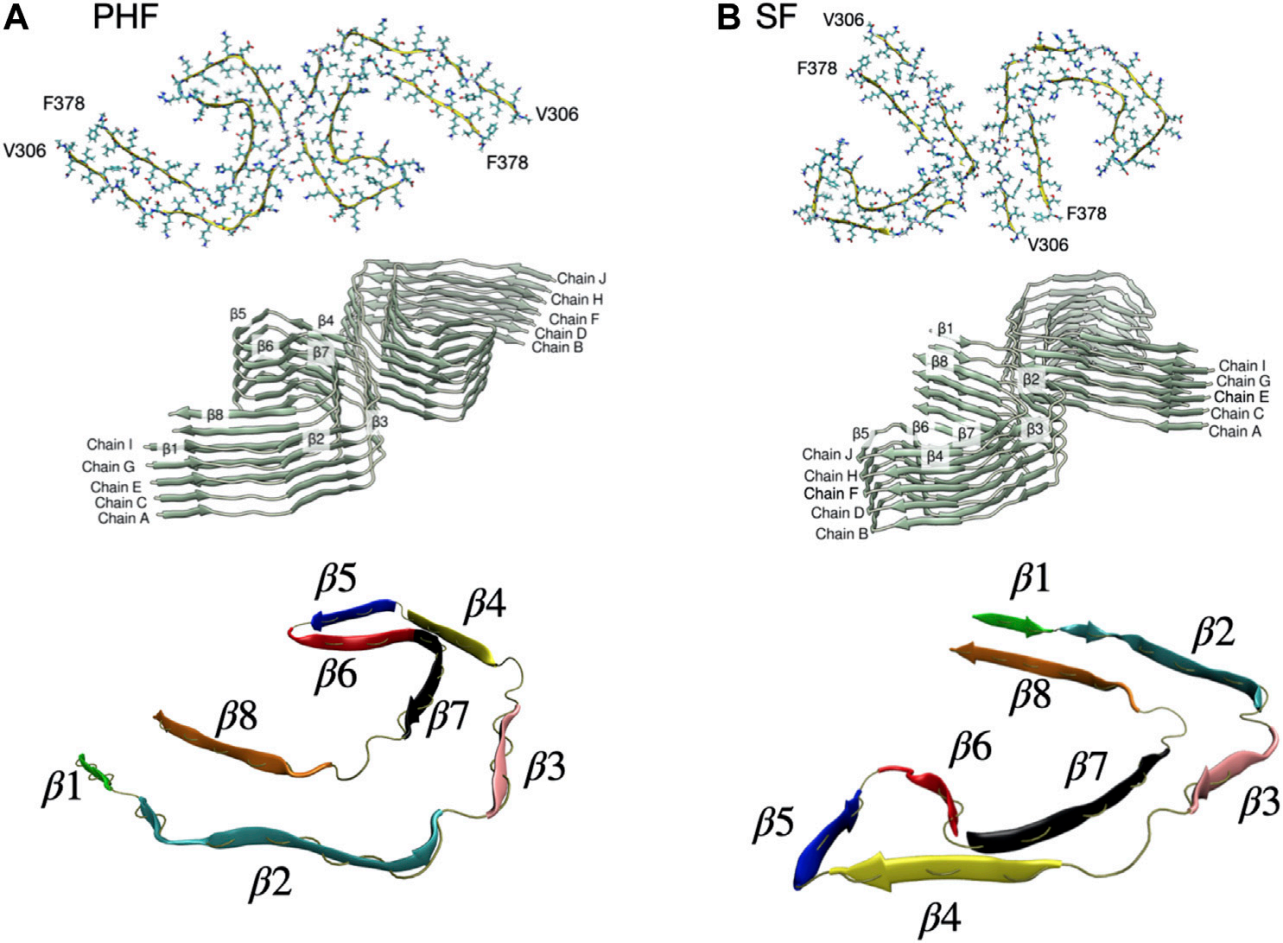
**(A)** PHF protofilament based on the cryo-EM R3-R4 structure of residues V306-F378 (PDB ID entry 503 L). We used ten total chains labelled A-J arranged as five paired filaments. The C-shaped core structure has eight β sheets labelled β1-β8. **(B)** SF protofilament based on the cryo-EM R3-R4 structure of residues V306-F378 (PDB ID entry 5O3T). The SF structure has the same eight β sheets labelled β1-β8 but a different orientation of paired filaments. A rendered view of the secondary structure is shown at the bottom with the β sheets individually colored.

The complete cryo-EM structures of both the PHF (PDB ID entry 503 L) and SF (PDB ID entry 5O3T) fibril contain 14 pairs of chains arranged in a helical stack with a C-shaped cross-section. As a minimal stable protofibril starting structure, we used ten protofilament core chains (chains A-J) resulting in a structure with five stacked and paired C-shaped subunits, as shown in Figure 1. The N-terminus of each peptide was capped with an acetyl group (ACE) and the C-terminus was capped with a N-methionine group (NME) to give uncharged terminal ends. A post-translationally modified PHF protofibril was created using the PyTMs plugin for PyMOL (Warnecke et al., 2014; Schrödinger, LLL, 2015). We added a phosphate group (−2 charge) onto Ser 356 for chains labelled G and I in Figure 1A. All titratable amino acids were assigned a charge based on physiological pH. We used the CHARMM36m force field (Huang et al., 2017) with the TIP3P water model. CHARMM36 parameters are available for phosphorylated serine amino acids. Each protofibril chain was solvated in a box with periodic boundary conditions. The system was neutralized with counter ions to achieve a final salt concentration of 150 mM.

All simulations were performed using the Gromacs 2019.4 MD code (Hess et al., 2008; Lindahl et al., 2019) with an integration time step of 2 fs. Long-range electrostatics were calculated using the PME method (Essmann et al., 1995), and we used a cutoff radius of 1.0 nm for both real-space Coulombic and Lennard-Jones interactions. Equilibrium MD simulations of both the SF and PHF protofibril in solution show that the structure is stable for at least 10 ns (see Supplementary Figure S1).

The protofibril was placed in an elongated box with dimensions 15.8 × 8.7 × 25.7 nm for PHF and 12.4 × 12.5 × 25.7 nm for SF, as determined by the minimum image convention for periodic boundary conditions. After solvating the box with TIP3P water and counter ions, the PHF system contained 358,966 atoms and the SF had 402,600 atoms. Following a steepest descent energy minimization step, the system was equilibrated for 100 ps in an NVT ensemble at a temperature of 310 K using the velocity rescaling thermostat (Bussi et al., 2007) and position restraints on all heavy atoms. This was followed by a 100 ps NPT equilibration using a Berendsen weak-coupling barostat (Berendsen et al., 1984) to maintain a pressure isotropically at 1 bar. Position restraints were removed from all heavy atoms except for peptides G and H, which were used as an immobile reference (See Figure 1 for a definition of chains and secondary structure). This restraint mimics the effect of the larger fibril structure (Takeda and Klimov, 2009a; Takeda and Klimov, 2009b). All production runs were performed in the NPT ensemble at a temperature of 310 K and pressure of 1 bar, using the velocity rescaling thermostat and the Parrinello-Rahman barrostat (Parrinello and Rahman, 1981).

To quantify structural changes that occur during a simulation, the distance root mean square deviation (dRMSD) of the backbone atoms with respect to a reference β sheet structure was monitored for each inter-chain β sheet formed between chain G and chain I (β1–β8 in Figure 1). The dRMSD was computed during the simulation using the PLUMED2.6 plugin (Tribello et al., 2014). The dRMSD is a measure of the distance between two structures $X^{a}$ and $X^{b}$, defined as

$$d\left(X^{A},X^{B}\right)=\sqrt{\frac{1}{N\left(N-1\right)}\underset{i\neq j}{\sum }{\left[d\left(x_{i}^{a},x_{j}^{a}\right)-d\left(x_{i}^{b},x_{j}^{b}\right)\right]}^{2}}$$ (1)

where *N* is the number of backbone atoms in the reference structure, and $d\left(x_{i},x_{j}\right)$ is the distance between atoms i and j. For each of the β sheets (β1–β8) the cryo-EM structure after a short 200 ps equilibration was used as the reference structure in the calculation.

### 2.1 Steered MD Simulations

For each SMD (COM pulling) simulation, the COM of peptide chain I was pulled away from the COM of peptide chain G of the fibril core along the *z*-axis for 1,200 ps using a constant pull rate of 0.01 nm ps^−1^ and a spring constant of 1,000 kJ mol^−1^ nm^−2^. The simulation box, placement of the chain, and definition of the elongation axis are shown in the Supplementary Figure S2. A slower pulling rate of 0.0025 nm ps^−1^ resulted in similar force curves (see Supplementary Figures S3–S5). A final COM distance between peptide chain I and the fibril end of approximately 10–12 nm was achieved.

### 2.2 Metadynamics Simulations

The dissociation path of a single tau peptide chain from the protofibril structure was investigated with metadynamics (Laio and Parrinello, 2002). In this work, the history-dependent metadyanimcs bias is applied in order to explore the dissociation pathway and identify partially folded intermediates. However, due to the slow convergence of the metadynamics bias, we do not compute the free energy surface from reweighting. Instead, we take representative configurations along the dissociation path sampled via metadynamics and perform umbrella sampling along the COM separation distance. Metadynamics enhances the exploration of phase space by applying a history-dependent bias potential along a chosen set of collective variables (CVs) (Valsson et al., 2016). Analogous to protein folding (Best et al., 2013), we compute the fraction of native contacts Q between chain I and the adjacent chains H, F, and G (see Figure 1). Here, Q includes all the native contact pairs i, j between heavy atoms i and j, considered in contact if the distance between i and j is less than 0.45 nm. The CV Q is then computed as

$$Q\left(X\right)=\frac{1}{N}\underset{\left(i,j\right)}{\sum }\frac{1}{1+\text{exp}\left[\beta \left(r_{ij}\left(X\right)-\lambda r_{ij}^{\circ }\right)\right]}$$ (2)

where $r_{ij}\left(X\right)$ is the distance between atom i and j in configuration X, $r_{ij}^{\circ }$ is the reference distance in the cryo-EM structure, β is a smoothing parameter set to 50 nm^−1^ and *λ* is a tolerance distance set to 1.5 nm. We identify 1,260 contacts between the terminal chain I and adjacent chains (G, H, and J) in the PHF structure and 1,149 contacts for the SF protofibril. The fewer native contacts in the SF structure is due to the looser packing of this structure and less contact between the adjacent paired helical structures shown in Figure 1.

Metadynamics simulations were carried out using the open-source, community-developed PLUMED library (Bonomi et al., 2019), version PLUMED2.6 plugin (Tribello et al., 2014). In addition to the fraction of native contacts CV Q, we also biased the COM distance between chains I and G (see Figure 1 for chain definitions). The two-dimensional bias was deposited every picosecond with a Gaussian hill height of 1.0 kJ/mol and a width of σ=0.01 for Q and σ=0.1 nm for the COM distance. Finally, a ratchet-and-pawl like restraint was placed on the COM distance to evolve the system toward further separation distances and dampen fluctuations back towards the protofibril end. A definition of this restraint is presented in the Supplementary Material Section 1.

### 2.3 Umbrella Sampling

From representative frames of the exploratory metadynamics trajectories, configurations were selected to generate starting configurations for umbrella sampling (Lemkul and Bevan, 2010). Frames for the umbrella sampling windows were selected every 0.1 nm up to 2 nm COM separation distance between chains I and G and every 0.2 nm beyond up to 11.0 nm. This resulted in a total of 62 umbrella windows for each protofibril. Since each umbrella window is taken from a snapshot of the metadynamics simulation, each window has the same box size and number of particles. For each umbrella window a harmonic restraint was employed centered at the reaction coordinate of the initial COM distance for that window. We used a force constant of 1,000 kJ mol^−1^ nm^−2^. After a short 200 ps equilibration, a 10 ns long production MD simulation was run for each umbrella window. The total production simulation time for the set of umbrella sampling simulations is 620 ns for each protofibril. A histogram of the sampled reaction coordinate for each system is shown in the Supplementary Figure S6, showing the overlap of the sampled distance distribution between adjacent windows. The free energy surface from the umbrella sampling simulations was computed using the weighted histogram analysis method (WHAM) as implemented in GROMACS 2019.4 (Hub et al., 2010). All umbrella sampling simulations were preformed on a GPU workstation (8 CPU threads and 1 Nvidia RTX 2080 GPU). We also made use of the SDSC Comet Supercomputer available through the Extreme Science and Engineering Discovery Environment (XSEDE) (Towns et al., 2014).

## 3 Results

In the present work we focus on the fibril stability of the two AD tau cryo-EM structures: the PHF filament and the SF filament. We also investigate a post-translationally modified PHF fibril with a phosphate group at Ser356, located in the MTB repeat domain. The PHF and SF filaments are structural polymorphs with the same number of amino acids but different packing and relative orientation of paired filaments. These structural differences are expected to lead to different relative stabilities of the two fibril types. As shown in Figure 1, the structural core unit for both fibrils consists of eight β-strands (labelled β1–β8) adopting a C-shaped structure.

### 3.1 SMD Simulations Identify That Interchain Contacts Formed by Residues Within the β6 and β7 Regions Impart Critical Structural Stability to the Protofibril

SMD simulations can be used to identify important interactions between subunits that are broken during the non-equilibrium trajectory (Izrailev et al., 1999). SMD has been applied in the context of protein-ligand binding (Grubmüller et al., 1996), DNA-binding proteins (Jakubec and Vondrášek, 2020), Aβ fibril growth (Lemkul and Bevan, 2010), and on tau fibril dissociation (Liu et al., 2019). During the non-equilibrium simulation, the force increases as a result of the applied bias until a breaking point is reached, at which time critical interactions are disrupted, allowing the peptide to dissociate from the core protofibril structure. The point of maximum force corresponds to the instant just before these key interactions are broken. Because the work performed during a SMD simulation is path-dependent, a single SMD pulling trajectory is insufficient to determine the free energy surface, and different force-time curves will produce different dissociation pathways. For this reason, the precise order of events leading to dissociation cannot reliably be determined from SMD simulations at the high force values used here.

**FIGURE 2:**
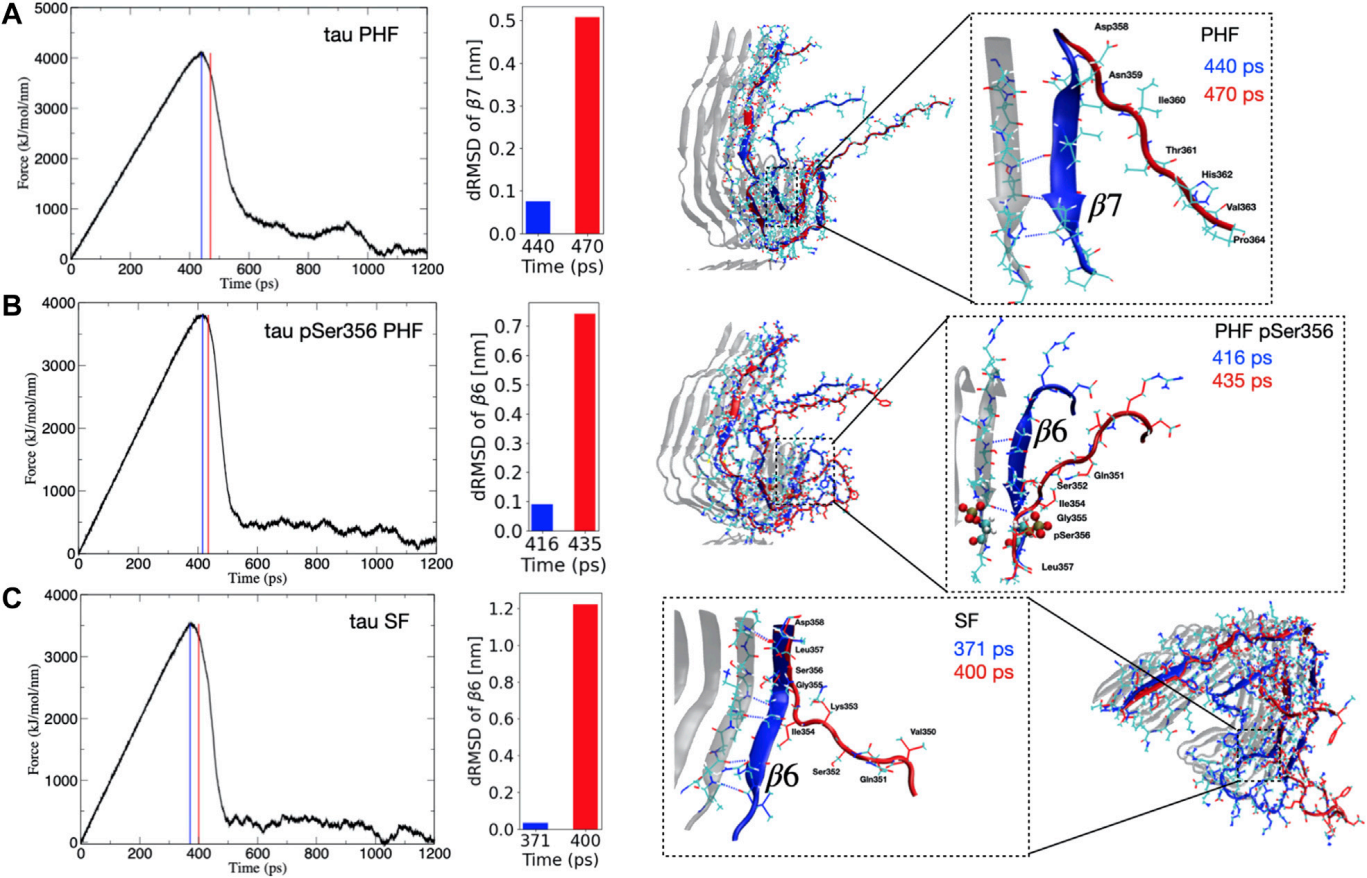
**(Left)** Time evolution of the force during SMD simulations for the **(A)** PHF, **(B)** phosphorylated pSer356 PHF, and **(C)** SF tau protofibrils. The blue vertical line (and depicted blue peptide structure on the right) corresponds to the structure at the maximum force. The vertical red line (and depicted red peptide structure on the right) correspond to a representative frame just after this maximum force has been reached and the force curve begins to decrease. This corresponds to a major structural transition where important hydrogen bonds have been broken during dissociation. **(Right)** The inset highlights the region of secondary structure loss that occurs between the point of maximum force (blue) and immediately after the force curve begins to decrease (red). For PHF **(A)** this event correlates with loss of the β7 secondary structure as indicated by the increase in the dRMSD value for this structure during this time window. For the PHF-pSer356 **(B)** and SF **(C)** the adjacent β6 region unfolds during the time window with corresponding increase in the dRMSD with respect to the reference β6 structure.

Despite the fact that tau dissociates from each protofibril end through different pathways, we consistently observe that the point of maximum force for each protofibril architecture corresponds to the breaking of hydrogen bonds between the parallel β-sheets, formed largely by residues Arg349 to Val363 that make up the β6–β7 region (see Figure 1). The observation that these hydrogen bonds break at the point of maximum force along the SMD trajectory suggests that these interactions impart critical stability to the protofibril. Figure 2 shows the force applied during the pulling of a single tau peptide chain from the protofibril tip during a 1,200 ps SMD simulation. A snapshot along the dissociation pathway at the point of maximum force is shown with the dissociating peptide chain colored blue, and the subsequent loss of structure immediately after the point of maximum force is shown with the dissociating peptide colored red. For the PHF protofibril (Figure 2A), the point of maximum force occurs at 440 ps and involves breaking the interchain hydrogen bonds between residues Ile360 and Val363 within the β7 region. A snapshot highlighting this region of the protofibril just before the point of maximum force at 440 ps is shown in blue and is compared to the same chain when the force-time curve is decreasing at 470 ps shown in red. We observe that during this 30 ps window, the *β*-sheet formed by residues Asp358-His362 (β7 in Figure 1) is lost and hydrogens bonds between parallel *β*-sheets in this region are broken. This is confirmed by the large increases in the distance dRMSD for the β7 region during this time window.

For the post-translationally modified PHF at Ser356 (PHF-pSer356), the force-time curve (Figure 2B) exhibits a maximum at 416 ps and involves loss of structure and breaking of interchain hydrogen bonds between the *β* sheet formed by residues Gln351 to Gly355 in the β6 region. The middle structure in Figure 2B compares a snapshot of the PHF-pSer356 structure just before the maximum force at 416 ps and at 435 ps when the force is decreasing, showing the interchain hydrogen bonds that are broken during this window. This region is directly adjacent to the phosphorylated Ser356. The dRMSD of the β6 sheet shows an abrupt increase during this time window indicating a loss of the β6 structure at the point of maximum pulling force.

For the SF protofibril (Figure 2C), the point of maximum force occurs earlier, at 371 ps. The structure just before the point of maximum force at 371 ps and at 400 ps (where the force is decreasing) is compared in bottom structure in Figure 2C, showing loss of the β-sheet structure between residues Gln351 and Asp358 that make up the β6 region and beginning of the β7 region. Interchain hydrogen bonds within this region are broken during this time window, and the dRMSD of the β6 sheet increases sharply as the β6 structure is lost.

In all SMD simulations, it is clear that a single main structural transition corresponds to a destabilization of the fibril structure, leading to dissociation at the protofibril tip. These structural transitions involve the parallel *β*-sheets that define the β6 and β7 region.

### 3.2 The Free Energy Surface From Umbrella Sampling Reveals Different Template-Induced Folding Mechanisms of Tau

Because the large pulling force may result in an unfolding mechanism that does not resemble the true dissociation path, we produce configurations along the dissociation path using metadynamics. Metadynamics builds a history-dependent bias during the simulation that allows the system to escape free energy minima, and the dissociation path revealed during a metadynamics simulation should closely follow the true dissociation mechanism. Furthermore, by including the fraction of native contacts Q as a CV, the applied bias should lead to enhanced fluctuations of native contacts leading to tau dissociation along the protein folding pathway. As the metadynamics bias builds during the simulation, weaker contacts should break before stronger contacts; thus the sequence of dissociation events from metadynamics should reflect the dissociation mechanism. We then perform umbrella sampling simulations restrained along the one-dimensional COM distance for frames extracted from metadynamics simulations.

**FIGURE 3:**
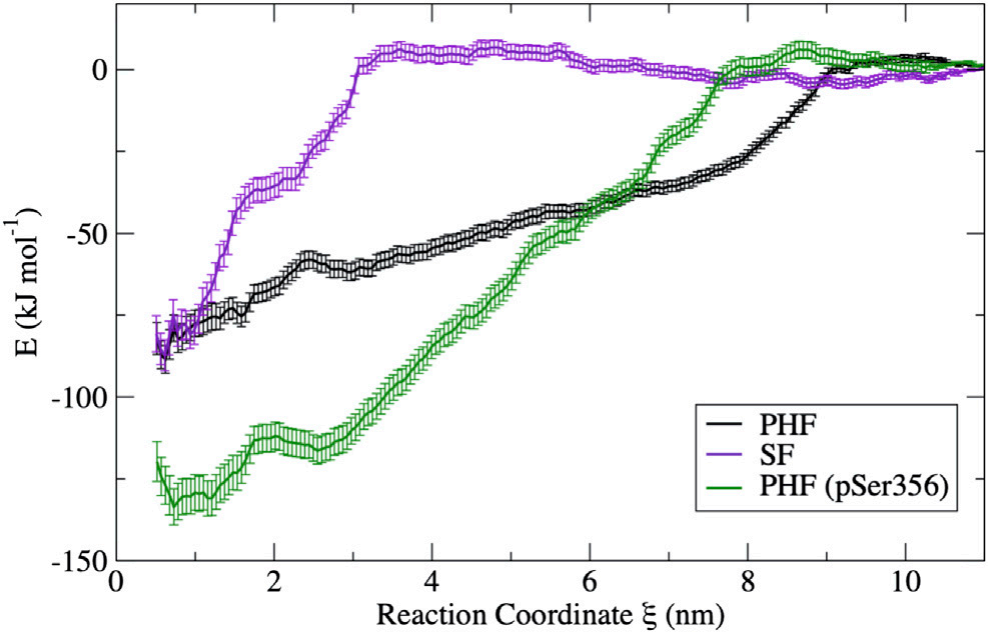
Free energy curves obtained from umbrella sampling simulations for each fibril in this study. The PHF free energy profile (black) and the SF free energy profile (violet) have a similar free energy minimum; however, the different shape reflects a different dissociation mechanism. The PHF fibril is stabilized by phosphorylation at Ser356 (green) despite having a similar mechanism of dissociation. Error bars were determined by bootstrapping.

The free energy surfaces computed from umbrella sampling simulations are shown in Figure 3. Error bars are determined using a bootstrap method (Hub and de Groot, 2006). Individual bootstrap profiles are presented in the Supplementary Figure S7. To provide a sense of the convergence of the free energy surface from the limited 10 ns umbrella sampling trajectories, the FES was computed separately for different trajectory blocks of 2–4, 4–6, 6–8, and 8–10 ns. A comparison of the FES for these different regions is presented in the Supplementary Figure S8. In all cases, the FES profile does not change appreciably after 5 ns. Therefore, the FES shown in Figure 3 is computed only over the final 5 ns of the 10 ns production simulation.

The difference in the free energy surfaces between the PHF fibril (black line) and SF fibril (purple line) of Figure 3 reflect differences in the dissociation and unfolding mechanism of tau for these two fibril structures. The PHF FES (black line) has a broader basin, indicating that partially folded tau forms contacts with the protofibril end at longer separation distances. The broad basin for the PHF fibril corresponds to the sequential breaking of interchain interactions, leading to unfolding of the tau peptide through a series of partially unfolded intermediates that will be discussed in more detail below. On the other hand, the FES for the SF fibril (purple line) has a narrower and steeper basin where partially folded tau makes contact with the protofibril end at shorter separation distance. The shape of the FES indicates that tau unfolds in a more concerted mechanism from the SF protofibril as compared to the PHF fibril. Interestingly, the difference in free energy between the bound structure (ξ=0.6 nm) and the free dissociated tau (ξ=11 nm) of both the PHF and SF protofibril is nearly identical, with a value of −89 kJ/mol ± 5 kJ/mol $\left(35\;k_{B}T\right)$ for the PHF and −87 ± 6 kJ/mol $\left(34\;k_{B}T\right)$ for the SF. This value compares reasonably well to the experimental value for fibril elongation of Aβ(1–40) of −38 kJ/mol $\left(34\;k_{B}T\right)$ (O’Nuallain et al., 2005). However, we note that our calculated value may include errors from limited sampling.

**FIGURE 4:**
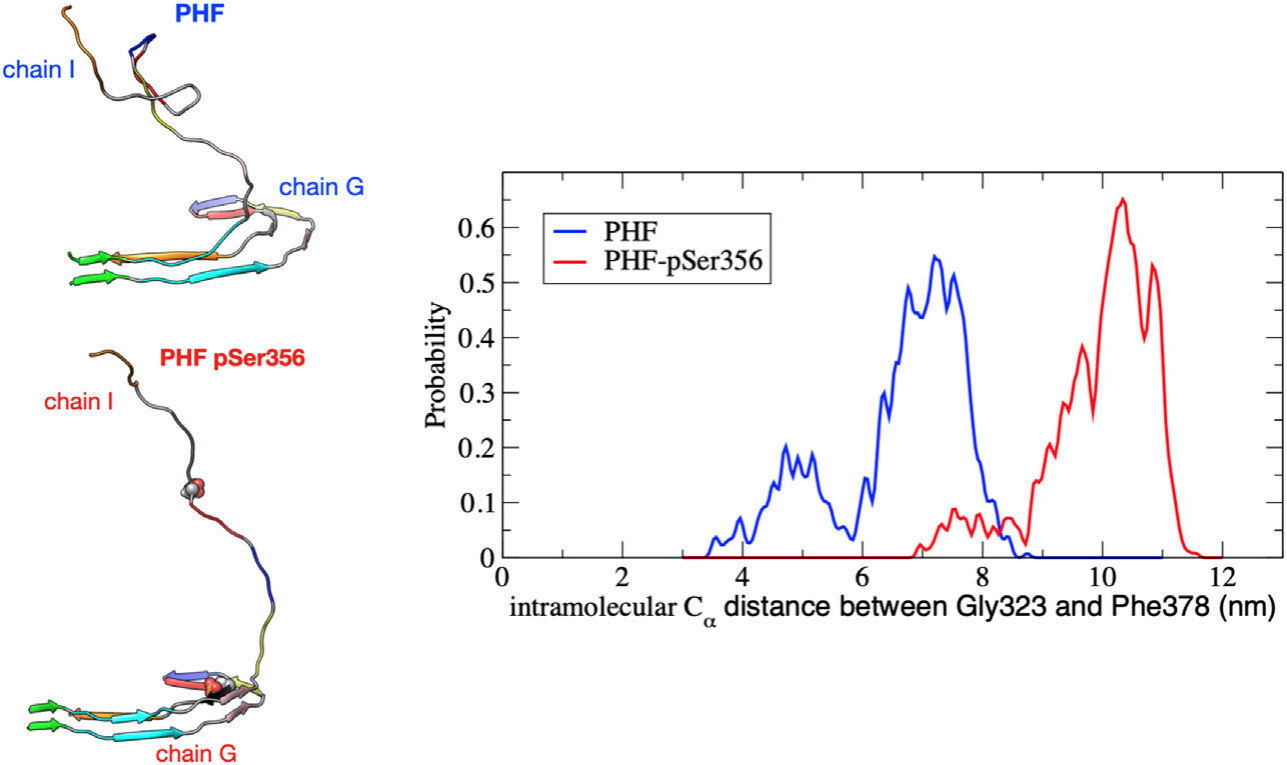
Distribution of the intramolecular Cα distance between residues Gly323 and Phe378 on chain I for the PHF (blue) and PHF-pSer356 (red) protofibril over the umbrella window restrained at a COM distance of 3.6 nm. The distribution is accumulated over the 10 ns production simulation. At this separation distance, the partially folded tau remains in contact with the protofibril at the β1 end (V306). A snapshot taken at 5 ns during the 10 ns trajectory is shown for both structures. The PHF-pSer356 remains predominantly in an extended conformation while the wild-type PHF adopts more compact configurations forming non-native intramolecular contacts.

Phosphorylation at pSer356 changes the shape of the FES profile for the PHF protofibril (green line). Interestingly, the free energy of the final folded state of the phosphorylated tau is lower with respect to the unphoshorylated PHF implying that the phosphate actually stabilizes the fibril structure. The origin of this increased stability of the pSer356 PHF protofibril is not obvious. Simulations of native tau in solution suggest that phosphorylation of tau at Ser356 can facilitate aggregation by destabilizing compact configuration and enhancing the distribution of extend conformations that expose residues to the protofibril template (Popov et al., 2019). To investigate this, Figure 4 shows a representative structure at a separation distances of ξ=3.6 nm for the PHF and PHF pSer356 along with the intramolecular distribution of the distance between the Cα of residues Gly323 and Phe378 on chain I averaged over the 10 ns trajectory. The wild-type tau adopts partially folded compact intermediate states while the phosphorylated tau remains in a much more extended conformation. As seen in Figure 3 (black line), after the wild-type tau makes initial contact with the PHF fibril at around 8 nm, the FES exhibits a docking region without a steep folding funnel. This feature agrees with recent models of amyloid aggregation progressing via a random search through multiple, non-productive conformations before the peptide samples an extended configuration that is able to form native contacts with the fibril template (Jia et al., 2017; Jia et al., 2020). In contrast, phosphorylation at Ser356 shifts the conformational ensemble towards more extended conformations (Figure 4), and the FES in Figure 3 (green line) exhibits a steeper folding funnel, along which native contacts form in successive order along the fibril template.

#### 3.2.1 The Dissociation Mechanism of Tau From the PHF Protofibril

**FIGURE 5:**
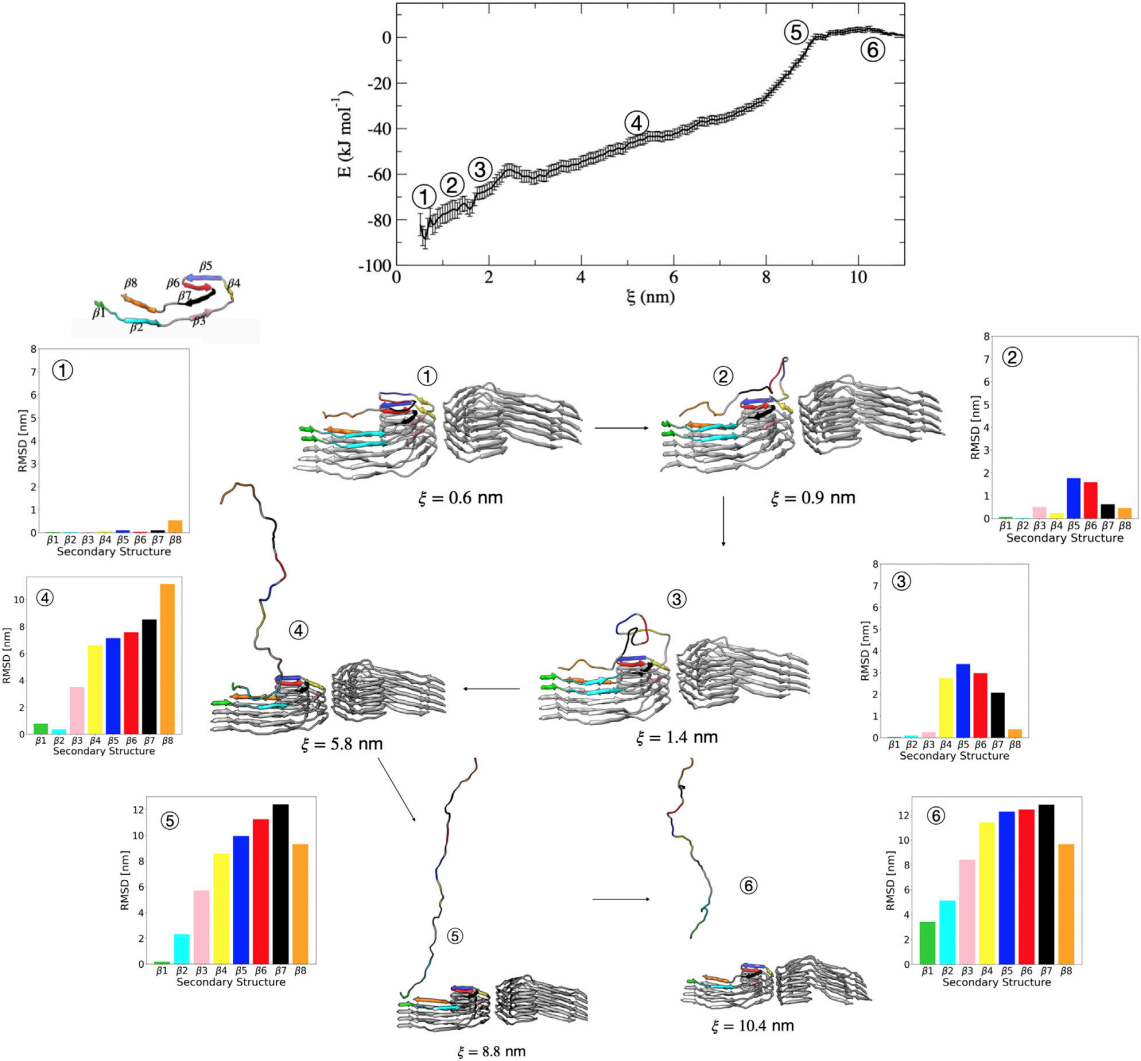
Free energy profile for the association/dissociation of a single tau chain onto the PHF protofibril. Representative structures are shown along with the dRMSD of the secondary structure regions. Between structures 1 and 2 the dRMSD increases for β5 (blue), β6 (red), and β7 (black). Structure 3 shows loss of β4 (yellow), and structure 4 shows loss of the β3 (pink). Structure 5 shows loss of β2 (cyan). The complete dissociation occurs with loss of β1 (green) that includes the PHF6 region. Structures were obtained from umbrella sampling simulation window within the different regions along the reaction coordinate after 5 ns. The dRMSD values in each of the bar graphs are calculated from Eq. 1 for the depicted representative structures taken at 5 ns of simulation, where the final folded state is the reference structure.

We now discuss in more detail the order of partial unfolding events that lead to dissociation of a single tau peptide from the PHF protofibril end. We analyze conformations from different umbrella windows along the reaction coordinate in terms of the dRMSD of the various β sheets that form the folded structure. Figure 5 shows representative structures from umbrella sampling windows at key intermediate stages of dissociation. Escape from the free energy minima that represents the bound conformation begins with loss of the β6 and β5 structure, followed almost immediately with partial stabilization of the β7 region. This conformational change is shown between structure 1 (ξ=0.6 nm) and structure 2 (ξ=0.9 nm) in Figure 5 and the corresponding increase in dRMSD for the β5-β7 region. Next, the β4 structure is lost between structure 2 and 3 (ξ=1.4 nm). Between structures 3 and 4 (ξ=5.8 nm), the partially unfolded tau forms non-native intramolecular contacts like that shown in Figure 4. The remaining sequence of dissociation events is loss of β3 between structures 3 and 4, loss of β2 between structures 4 and 5 (ξ=8.8 nm), and finally, loss of β1 between structures 5 and 6 (ξ=10.4 nm) that results in complete dissociation (structure 6) of the peptide. This final dissociation step (loss of β1) involves breaking interactions between the nucleating PHF6 hexapeptide ^306^VQIVYK^311^ region. This supports the hypothesis that the formation of the PHF6 hexapeptide initiates tau misfolding and aggregation.

#### 3.2.2 Effect of Phosphorylation at pSer356 on the Dissociation Mechanism of Tau From the PHF Protofibril

**FIGURE 6:**
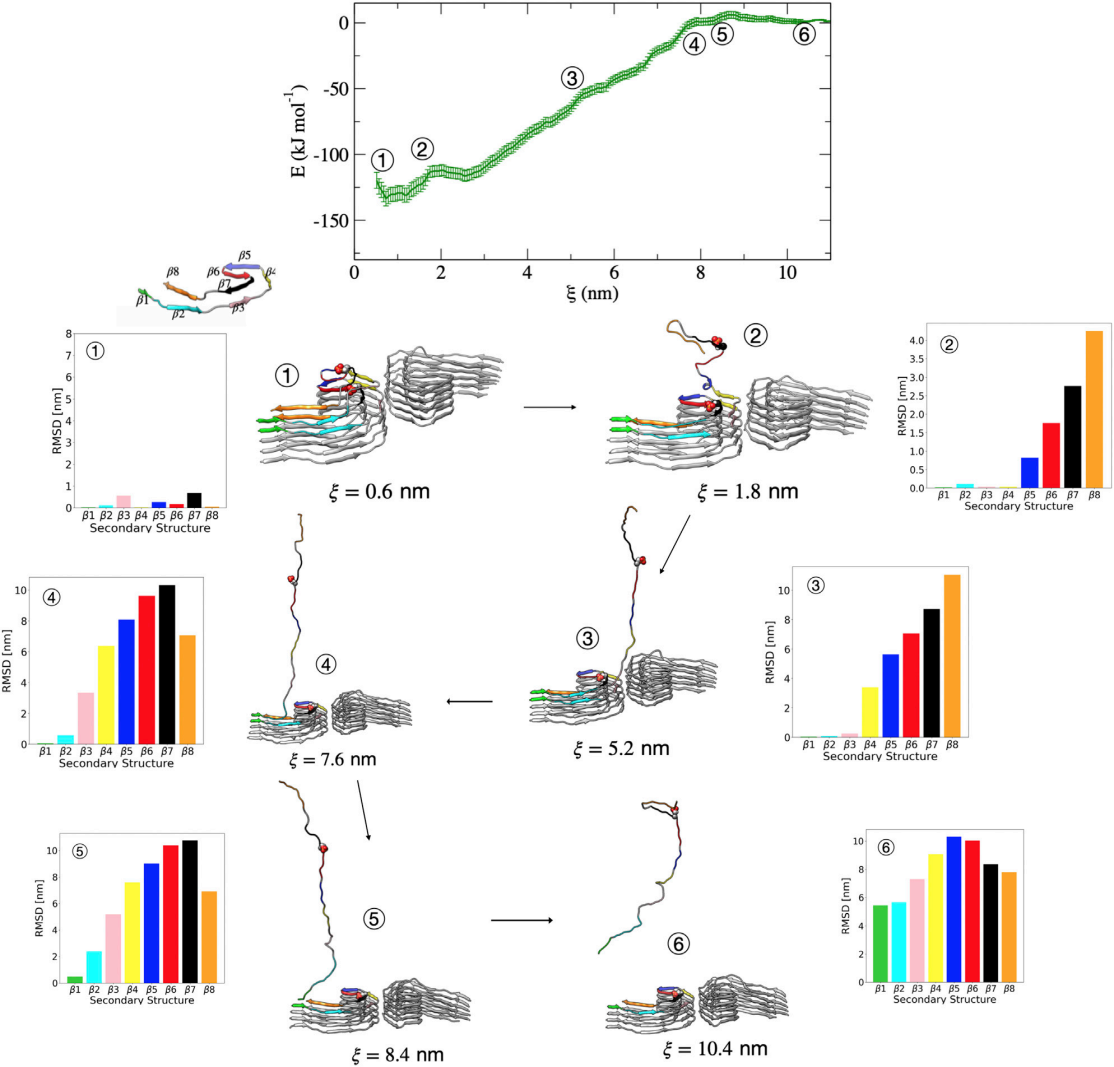
Free energy profile for the association/dissociation of a single pSer356 peptide from the phosphorylated PHF protofibril at Ser356. Representative structures are shown along with the dRMSD of the secondary structure regions. Between structures 1 and 2, the dRMSD increases for β5 (blue), β6 (red), β7 (black), and β8 (orange). Structure 3 shows loss of the β4 (yellow), and structure 4 shows loss of β3 (pink). Structure 5 shows loss of β2 (cyan). The complete dissociation occurs with loss of β1 (green) that includes the PHF6 region. Structures were obtained from umbrella sampling simulation window within the different regions along the reaction coordinate after 5 ns. The dRMSD values in each of the bar graphs are calculated from Eq. 1 for the depicted representative structures taken at 5 ns of simulation, where the final folded state is the reference structure.

Figure 6 shows representative structures along the reaction coordinate for the pSer356 post-translationally modified tau. The mechanism of dissociation in terms of the order of events is similar to that of the wild-type PHF. In this case, escape from the free energy minima that represents the bound conformation begins with loss of the β7 structure followed immediately by loss of the adjacent β6 and β8 region. This is shown in Figure 6 by the increase in dRMSD between structure 1 (ξ=0.6 nm) and structure 2 (ξ=1.8 nm) for these regions. Next, the β5 structure is lost, followed by the loss of β4 as shown between structure 2 and structure 3 (ξ=5.2 nm). The remaining unfolding events proceed identically to the wild-type PHF in the order of β4 → β3 → β2 → β1. We concluded that for both PHF and PHF pSer356 the β1-β4 region is involved in nucleation and the initial docking of tau to the protofibril template, occurring at separation distances between 2.0 and 9.0 nm. The subsequent folding of the β6-β8 regions leads to locking of tau into a pathological structure and occurs at separation distances less than 2.0 nm.

#### 3.2.3 The Dissociation Mechanism of Tau From the SF Protofibril

**FIGURE 7:**
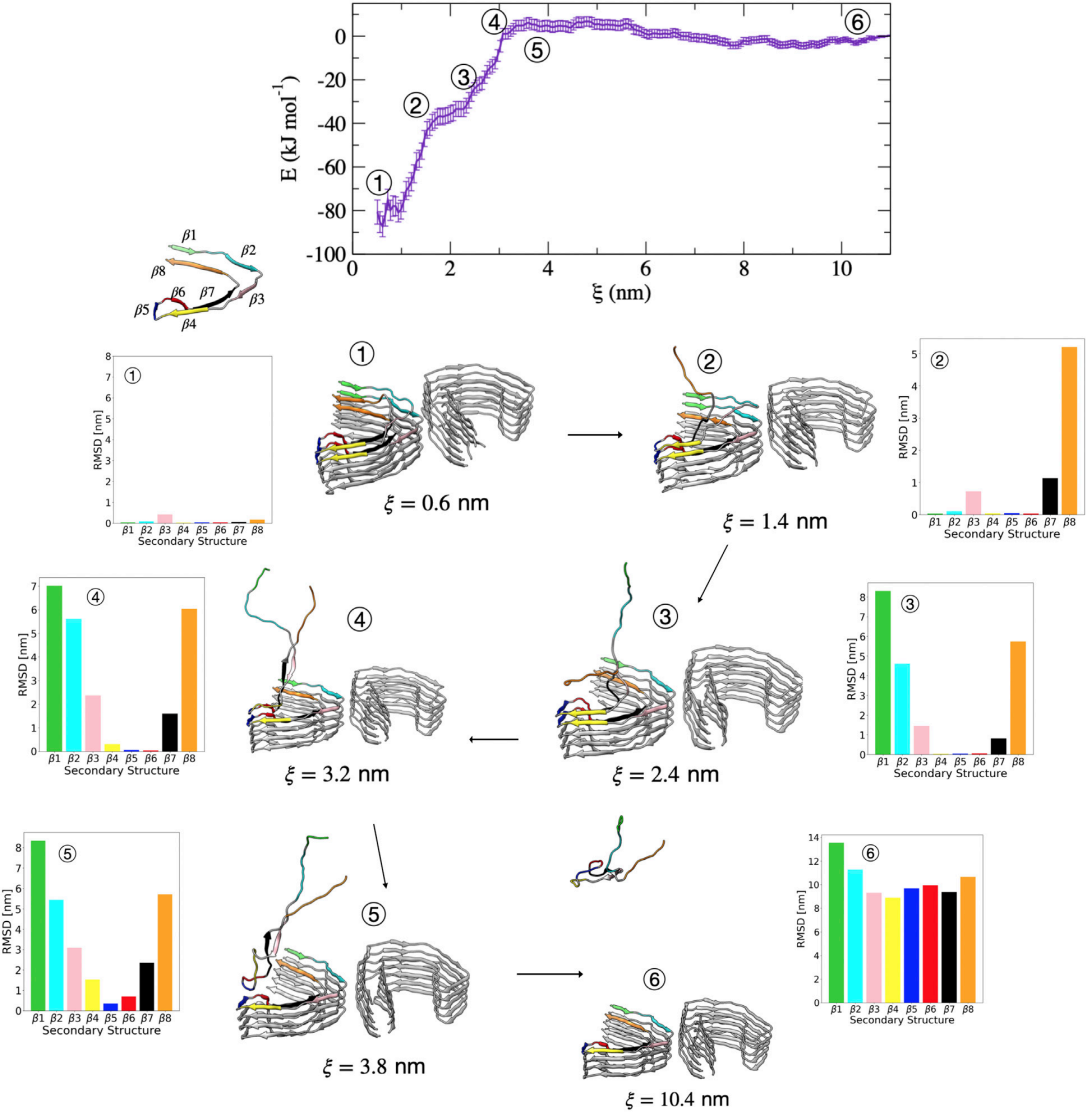
Free energy profile for the association/dissociation of a single tau chain onto the SF protofibril. Representative structures are shown along with the dRMSD of the secondary structure regions. Tau dissociates starting from the β8 end, shown by the increase in dRMSD between structures 1 and 2 for β7 (black) and β8 (orange). Structure 3 shows simultaneous loss of β1 (green) and β2 (cyan). Structure 4 shows simultaneous loss of β3 (pink) and β7 (black). Structure 5 shows loss of β4 (yellow) and an increased dRMSD for β6 (red). The complete dissociation occurs with loss of β5 (blue). Structures were obtained from umbrella sampling simulation window within the different regions along the reaction coordinate after 5 ns. The dRMSD values in each of the bar graphs are calculated from Eq. 1 for the depicted representative structures taken at 5 ns of simulation, where the final folded state is the reference structure.

Compared with the PHF protofibril, tau dissociates from the SF protofibril with a different unfolding mechanism, as suggested by the different free energy surface. Figure 7 shows representative structures from umbrella sampling windows at key intermediate stages of dissociation of a single tau peptide from the SF protofibril end. Dissociation begins with loss of the β8 region, as shown by the increase in dRMSD for this region between structures 1 (ξ=0.6 nm) and 2 (ξ=1.4 nm) shown in Figure 7. In contrast to the PHF, the β1 and β2 regions unfold next, as shown by structures 2 and 3 (ξ=2.4 nm), followed by the loss of β7 (between structures 3 and 4 (ξ=3.2 nm)). Between structures 4 and 5 (ξ=3.8 nm) along the FES, loss of the β4 and β6 region occurs. The final dissociation step involves loss of the β5 region between structures 5 and 6 (ξ=10.4 nm).

## 4 Discussion

Using all-atom MD simulations and a combination of enhanced sampling methods including non-equilibrium SMD, metadynamics, and umbrella sampling, we have determined several key factors that are important for the stability of tau AD NFTs and for fibril elongation through induced folding of monomeric tau. Our computational study suggests that the β6–β7 region imparts stability to both the PHF and SF fibril despite differing dissociation mechanisms. SMD simulations indicate that intermolecular contacts formed within this region are energetically strongest. We explore the full dissociation mechanism using metadynamics simulations, enhancing fluctuations along both the COM distance and the fraction of native contacts formed with the fibril end. We observe a different dissociation mechanism between the PHF and SF protofibril. Using umbrella sampling, we compute a free energy surface of tau dissociation from the protofibril end along the COM distance coordinate.

Assuming that fibril elongation proceeds via the reverse process of the observed dissociation, metadynamcis simulations show that elongation of the tau PHF fibril begins with association at the β1 and β2 region. The β1 region, including the PHF6 (^306^VQIVYK^311^) motif, is important for nucleation and docking of bulk tau in solution to the PHF fibril. This observation is supported by previous simulations that identified the PHF6 region as a critical fragment for nucleation of amyloid structures (Ganguly et al., 2015). The folding of tau along the PHF fibril template then proceeds with the sequential formation of the β3, β4 and β5 regions. Finally, the formation of native contacts within the β6–β7 region locks the tau peptide at the fibril end. In contrast, tau in solution initiates contact with the SF fibril end at the β5 region, forming the β4–β6 region, followed by β3 and β7 formation, before the templated folding of the β1 and β2 region. Taken together, the free energy surface and corresponding key intermediate structures presents a detailed picture of important steps in AD pathogenesis. Such detailed mechanistic information can give insight into tau seeding experiments in which different protofibril seeds can templet different tau morphologies (Strang et al., 2018).

Post-translational modification or mutations that affect the stability of the fibril might disrupt the formation of toxic NFTs. We identify the β6–β7 region as being important for maintaining both the SF and PHF protofibril stability. To investigate this idea, we have studied a post-translationally modified PHF fibril phosphorylated at Ser356, which is located between the β6 and β7 region. Our results show that pSer356 modifies the FES and alters the order of the β6 and β7 loss of structure in the dissociation mechanism. However, it is not obvious how this subtle difference in the mechanism will manifest during *in vitro* tau seeding experiments. Phosphorylation of Ser356 has been shown experimentally to block tau interactions with Aβ peptide (Guo et al., 2006) and inhibits the seeding activity of the K18 tau construct in the presence of heparin (Haj-Yahya et al., 2020). Meanwhile, REMD simulations of the PHF dimer show that pSer356 modifies the conformational ensemble of a tau dimer in solution (Derreumaux et al., 2020). It has been suggested that pSer356 may lead to increased sampling of extended conformations of disordered tau, thereby exposing residues to the fibril template during binding (Popov et al., 2019). Our umbrella sampling simulations give credence to this idea, showing that partially folded pSer356 tau remains extended while docking and is able to form native contacts with the fibril template without needing to unfold compact conformations or break non-native contacts. Further experiments and simulation work is needed to fully understand the effect of phosphorylation at Ser 356 as well as other possible phosphorylation sites within the MTB region.

It would be interesting in the context of AD targeted therapeutics to investigate small molecule inhibitors or mutations that affect the β5–β7 region. This region is involved in the final folding of tau onto the PHF template, while the formation of initial contacts between tau and the SF fibril involves the β5 region. While the anthraquinone derivative Purpurin molecule has been shown to inhibit tau fibrillization by forming hydrophobic contacts with the PHF6 nucleating hexapeptide region ^306^VQIVYK^311^, our results suggest the β5–β7 regions as an alternative target.

This work presents a detailed thermodynamic and mechanistic analysis of tau fibril dissociation for the two structural polymorphs of tau relevant to AD neurodegeneration using recent cryo-EM structures. Differences in the FES for template-induced misfolding of tau by the two AD protofibril structures can provide a more complete understanding of tau seeding from these structures. In addition to further work to understand how mutations and the binding of small molecules might perturb the thermodynamics of fibril elongation through templated folding, simulations of other tau morphologies, such as the widely studied K18 construct, could give additional insights into tau folding mechanisms. While this work presents a picture of tau dissociation, the free energy surface projected along the one dimensional separation distance may hide other relevant conformations or missing slow degrees of freedom that could provide more thermodynamic insight. The present study could be complemented by other enhanced sampling methods that could more completely explore configuration space. Another area for further exploration is in the kinetics of dissociation of tau, which could be elucidated by studying the position-dependent diffusion along the reaction coordinate.

## Data Availability

The raw data supporting the conclusions of this article will be made available by the authors, without undue reservation.
