# Supplementary material for: Insight Into Seeded Tau Fibril Growth From Molecular Dynamics Simulation of the Alzheimer’s Disease Protofibril Core
Source: Front Mol Biosci. 2021 Mar 19;8:624302. doi: 10.3389/fmolb.2021.624302 (PMC8017153; doi:10.3389/fmolb.2021.624302)
Supplement: Supplementary file 1 [file datasheet1.pdf]

Supplementary Material

1 SUPPLEMENTARY METHODS

1.1 Definition of the ratchet-and-pawl like restraint

The ratchet-and-pawl like restraint is used to ensure the tau monomer dissociates away from the fibril end and is prevented from diffusing back and refolding at the fibril end. The bias uses a harmonic potential moving along with the thermal fluctuations of the COM distance between chain I and G. The bias potential has the form:

$$V(\rho(t)) = \begin{cases} \frac{K}{2}(\rho(t) - \rho_m(t))^2, & \rho(t) > \rho_m(t) \\ 0, & \rho(t) \leq \rho_m(t) \end{cases} \tag{S1}$$

where

$$\rho(t) = (\xi(t) - 12.0)^2 \tag{S2}$$

and

$$\rho_m(t) = \text{min}_{0\leq\tau\leq t}\rho(\tau) + \eta(t) \tag{S3}$$

We used a value of $K = 500$ kJ mol^{-1} nm^{-2} and $\xi(t)$ is the COM distance (measured in nanometers) between chain I and chain G at time t. $\eta(t)$ is an white noise acting on the minimum position of the bias.

2 SUPPLEMENTARY FIGURES

2.1 Figures

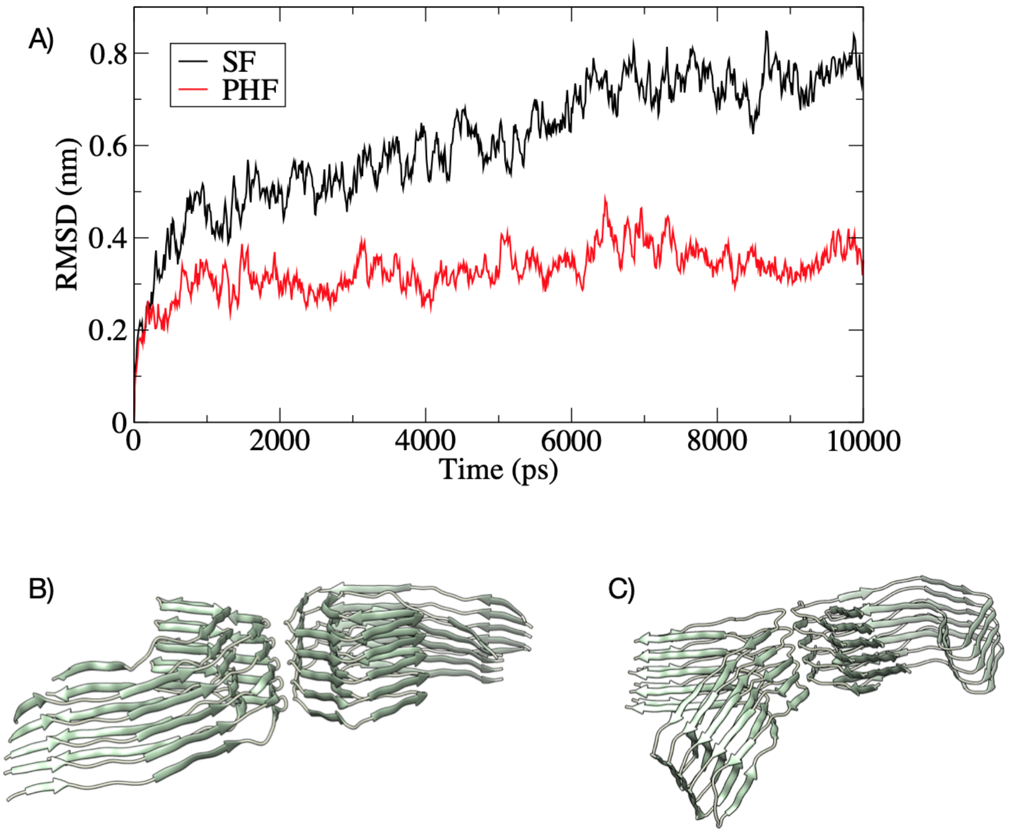

Figure S1. A) Root-mean-square deviation (RMSD) of the backbone atoms with respect to the starting structure during a 10 ns NPT MD simulation at 310 K and 1 bar for the PHF protofibril (red) and SF protofibril (black). The RMSD shows that the PHF structure quickly stabilizes around 0.35 nm while the SF structure deviates from the initial structure up to 0.8 nm. The final equilibrium configuration is shown for the PHF (B) and SF (C) protofibril. In both simulations the core protofibril structure remains intact and stable. The orientation of the paired filament chains for the SF structure changes more during the simulation than the PHF structure, which explains the larger RMSD value for the SF structure.

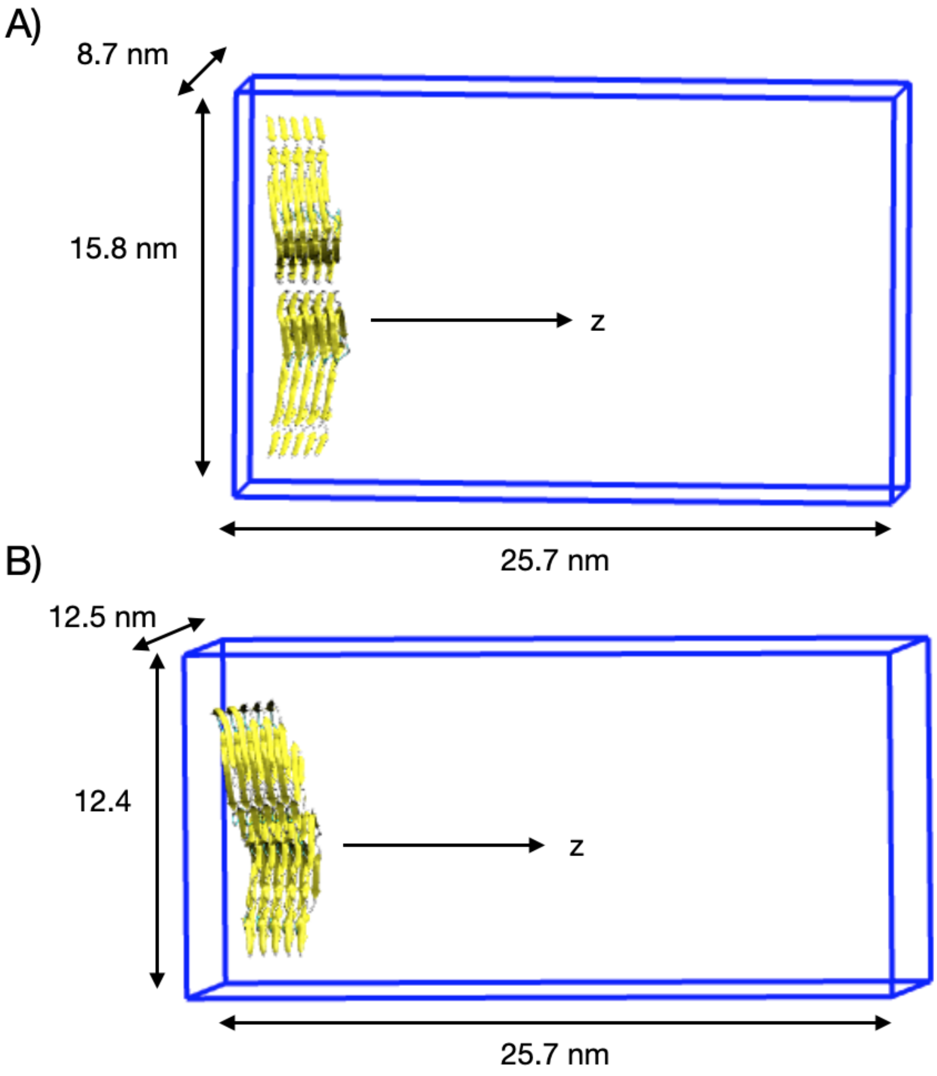

Figure S2. Simulation box setup and pulling direction for the SMD simulations of the (A) PHF protofibril and (B) SF protofibril. The box is elongated along the z-axis which is the pulling direction. The maximum final center of mass (COM) distance realized during the SMD simulation is 12 nm, which must be less than one half of the simulation box in the z-direction (here set to 25.7 nm). The simulation box size for the pSer356 PHF protofibril was identical to the PHF protofibril shown in A.

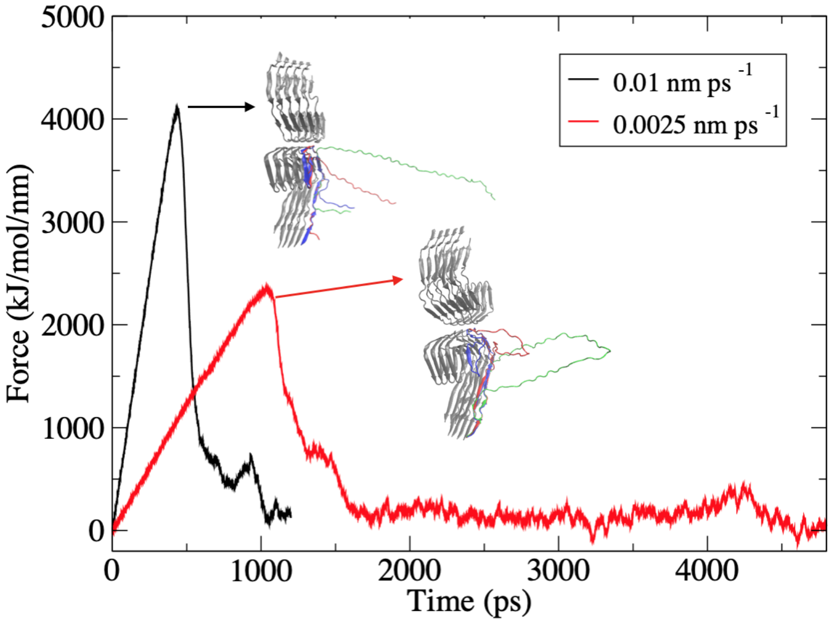

Figure S3. Comparison of the force vs. time over the SMD trajectory using a pulling rate of 0.01 nm ps^{-1} (black) and a pulling rate of 0.0025 nm ps^{-1} for the PHF protofibril. Both curves show one major structural transition that corresponds to the loss of the secondary structure of the $\beta6$ to $\beta7$ region. Both SMD simulations exhibit a similar dissociation pathways as shown by the representative snapshots, colored sequentially blue, red, and green.

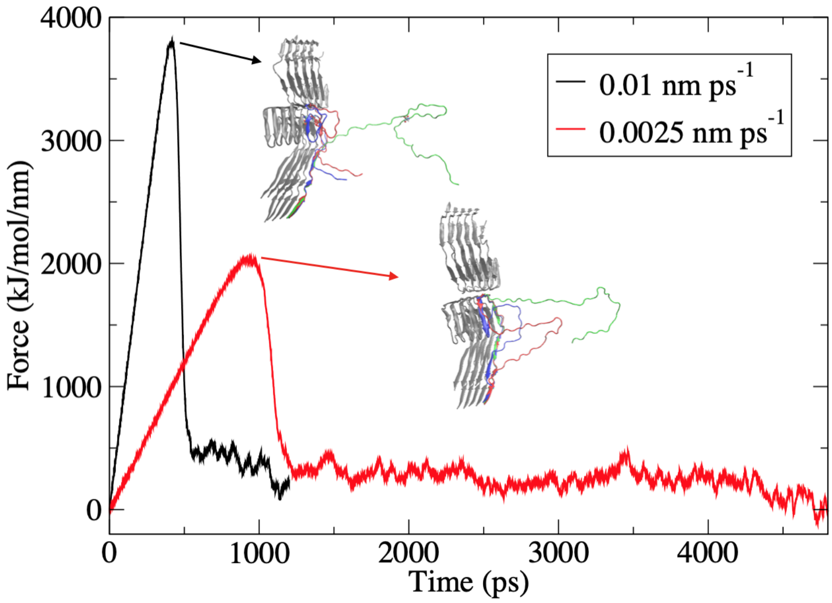

Figure S4. Comparison of the force vs. time over the SMD trajectory using a pulling rate of 0.01 nm ps^{-1} (black) and a pulling rate of 0.0025 nm ps^{-1} for the post-translationally modified PHF protofibril, pSer356. Both curves show a single major structural transition within the $\beta6$ region, flanking the phosphorylated Ser356. Both SMD simulations exhibit a similar dissociation pathways as shown by the representative snapshots, colored sequentially blue, red, and green.

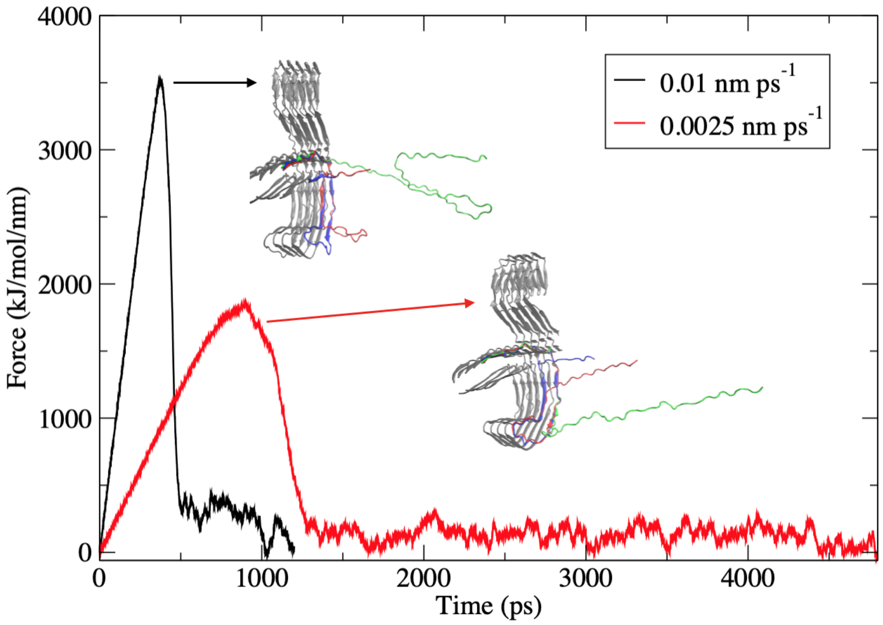

Figure S5. Comparison of the force vs. time over the SMD trajectory using a pulling rate of 0.01 nm ps^{-1} (black) and a pulling rate of 0.0025 nm ps^{-1} for the SF protofibril. Both curves show one major structural transition, corresponding to the loss of the secondary structure of the $\beta 6$ region. Both SMD simulations exhibit a similar dissociation pathways as shown by the representative snapshots, colored sequentially blue, red, and green.

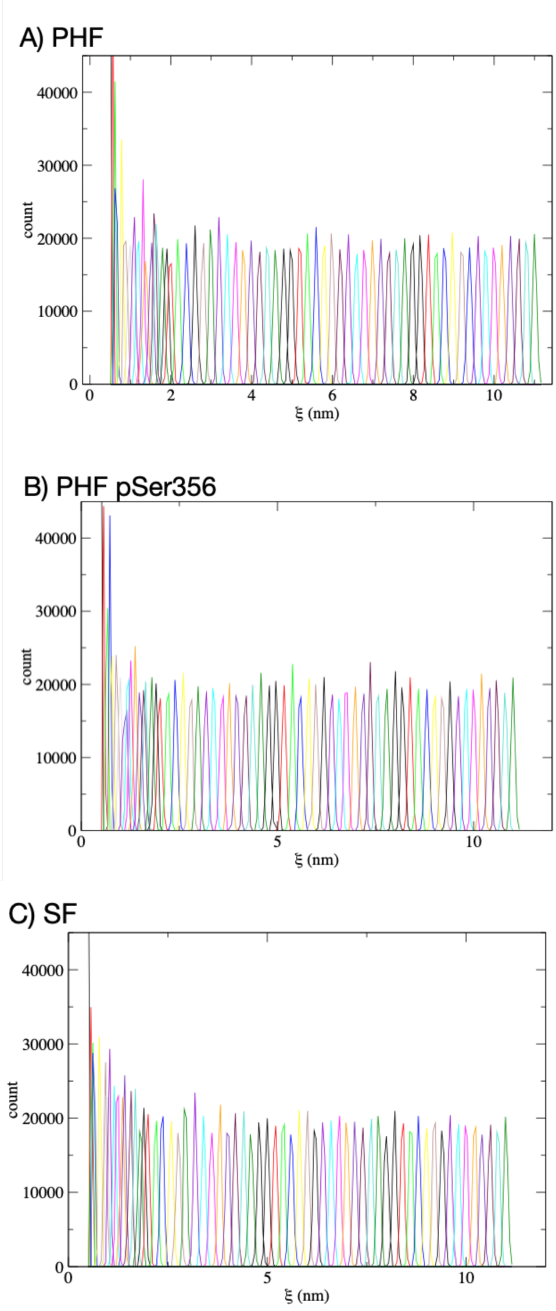

Figure S6. Histogram of the sampled distances along the reaction coordinate for each umbrella window for the (A) PHF, (B) pSer356 PHF, and (C) SF. Each system contained 62 umbrella windows. Windows were chosen from metadynamics simulation trajectories with a target spacing of 0.1 nm between windows up to 2 nm COM separation distance, followed by a spacing of 0.2 nm for COM distances above 2 nm. The histogram is computed for the final 5 ns of the 10 ns trajectory for each window.

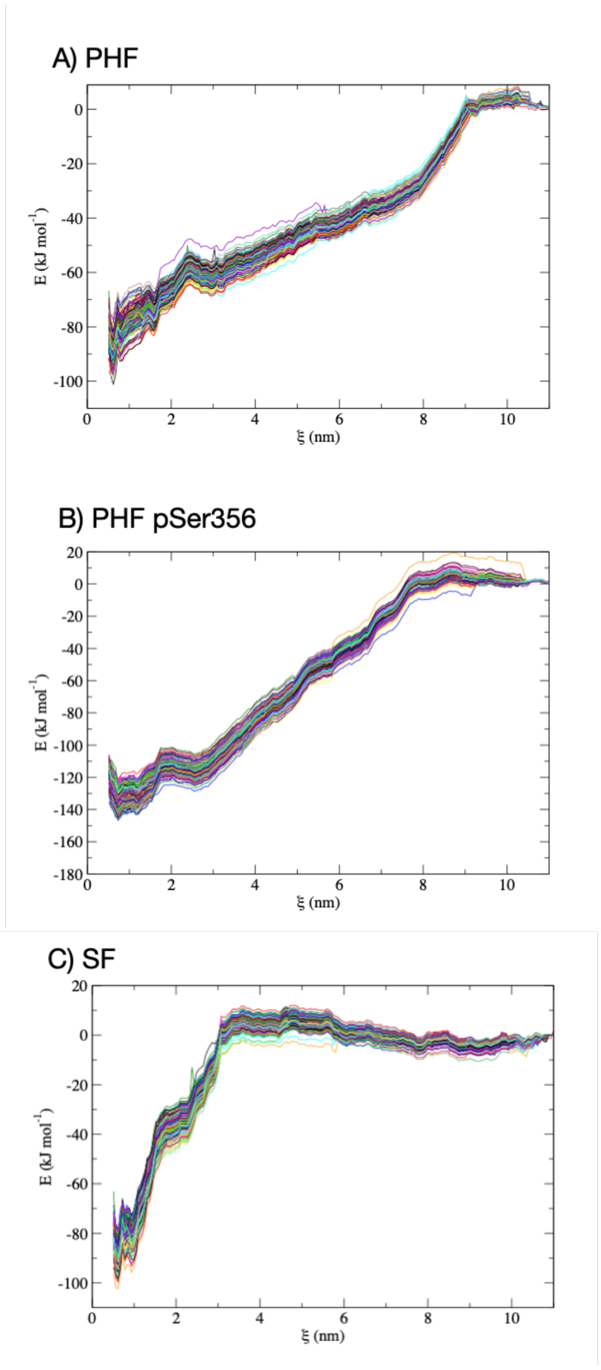

Figure S7. Bootstrap profiles for the free energy surfaces (FES) of (A) the PHF protofibril, (B) the pSer356 protofibril, and (C) the SF protofibril determined from umbrella sampling. We used 200 bootstrap samples in each case. The bootstrap profiles are used to estimate the error bars of the FES.

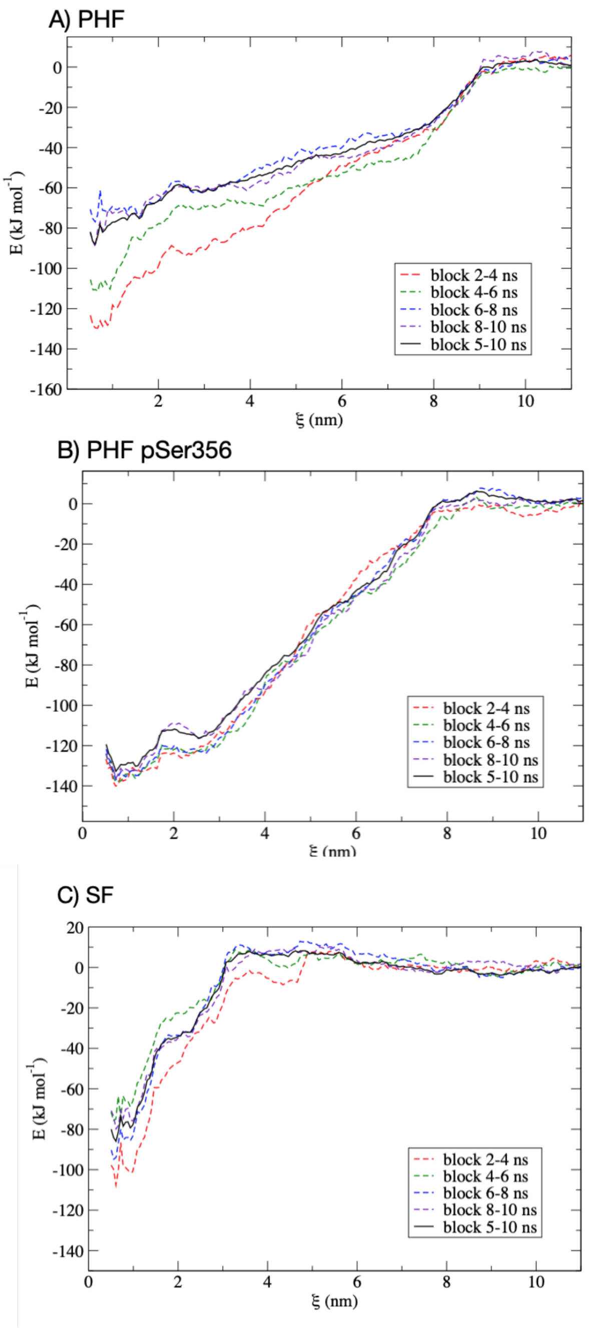

Figure S8. Convergence of the FES profiles. The total production time is 10 ns for each umbrella window. The first 2 ns of the trajectory is discarded for equilibration. Then, the FES is computed over different blocks of trajectory, using the time range 2-4 ns (red dashed line), 4-6 ns (green dashed line), 6-8 ns (blue dashed line), and 8-10 ns (purple dashed line). The solid black line shows the FES computed from the final 5 ns of the trajectory for each window. (A) For the PHF fibril, FES profiles are similar between 6-8 ns (blue dashed line) and 8-10 ns (purple dashed line) simulation blocks. Both the PHF pSer356 fibril (B) and SF fibril (C) show faster convergence and less variation between block as compared with the PHF. In all cases, the final 5 ns of the 10 ns production run for each window was used to generate the FES profiles presented in the main text.
